# Sexual Function, Medication-Related Motivation and Knowledge, and Perceived Spousal Support in Women with Eczema: A Cross-Sectional Study

**DOI:** 10.3390/healthcare14213785

**Published:** 2026-11-05

**Authors:** Dinçer Atila, Akgün Yeşiltepe, Semra Bulbuloglu, Vatan Barışık

**Affiliations:** 1 Family Health Center Number 1, Menemen 35000, Turkey; 2 Department of Midwifery, Faculty of Health Sciences, Munzur University, Tunceli 62000, Turkey; 3 Department of Nursing, Faculty of Health Sciences, İzmir Bakırçay University, İzmir 35000, Turkey; 4 Division of Surgical Nursing, Nursing Department, Faculty of Health Sciences, Istanbul Aydin University, Istanbul 34000, Turkey; 5 Department of Midwifery, Faculty of Health Sciences, Istanbul Aydin University, Istanbul 34000, Turkey; 6 İzmir Metropol Hospital, İzmir 35000, Turkey

**Keywords:** eczema, sexual dysfunction, medication adherence, spousal support, women’s health, antihistamines, dermatology

## Abstract

**Background:** Eczema may affect not only dermatological health but also sexual well-being, treatment-related behaviors, and interpersonal relationships. Examining sexual function alongside medication-related factors and perceived spousal support may provide a broader understanding of women’s experiences of eczema. **Methods:** This cross-sectional study aimed to evaluate sexual dysfunction, medication adherence, and perceived spousal support and to examine the relationships among these factors in women with eczema. The study included 218 women diagnosed with eczema who were regularly followed in the dermatology unit of a private hospital in İzmir, Turkey. Data were collected using a personal information form, the Modified Morisky Adherence Scale (MMAS), the Spousal Support Scale (SS), and the Arizona Sexual Experiences Scale (ASEX). Descriptive statistics, Pearson correlations, Spearman sensitivity analyses, and three-step hierarchical multiple linear regression were performed. **Results:** All participants were using antihistamines, and 71.6% reported using them twice daily. According to the ASEX cutoff score, 59.2% of participants screened positive for potential sexual dysfunction, while 56.9% reported not receiving spousal support. The mean ASEX score was 16.19 ± 8.94, and the mean MMAS score was 2.53 ± 2.21. ASEX scores were moderately and positively correlated with MMAS scores (r = 0.528, *p* < 0.001), whereas the SS total score was not significantly correlated with ASEX (r = −0.026, *p* = 0.704). ASEX scores were found to be positively correlated with scores on both the motivation (r = 0.568; *p* < 0.001) and knowledge (r = 0.426; *p* < 0.001) subdimensions. In hierarchical regression, MMAS scores remained positively associated with ASEX scores after adjustment for age, menopausal status, chronic disease, and antihistamine use duration, although the association was considerably weaker (B = 0.700, *p* = 0.038); this model explained 51.4% of the variance. Adding sexual intercourse frequency increased the explained variance to 70.5%, and the MMAS association was no longer statistically significant (B = 0.316, *p* = 0.237). **Conclusions:** Sexual dysfunction was common in this sample of women with eczema. In bivariate analyses, higher scores for medication-related motivation and knowledge were associated with greater sexual dysfunction. The association between total MMAS and ASEX scores remained significant after adjustment for clinical factors; however, this association weakened and lost statistical significance when sexual intercourse frequency was included in the model. While these findings support the need to consider sexual health during clinical assessment, they do not establish a causal relationship between medication adherence and sexual functioning.

## 1. Introduction

Eczema is a common inflammatory skin disorder affecting individuals of all ages, presenting with symptoms of vesiculation, pruritus, and erythema in its acute phase. The terms eczema and dermatitis are often used interchangeably to describe inflammatory skin conditions [1,2,3]. Since eczema weakens the barrier function of the skin, it causes the skin to become more sensitive. In individuals with severe eczema, cracking of the skin occurs, and fluid-filled blisters may form [4]. Protecting and restoring skin barrier function is an important component of eczema management. Dietary and environmental approaches have also been investigated, although their relevance depends on the clinical context and available evidence [5]. The global prevalence of eczema ranges between 10% and 20% [6]. Regarding female sexual health, studies conducted in Turkey reported FSFI-based sexual dysfunction rates of 53.2% in a sample of healthy women and 39.1% in a sample assessed during the COVID-19 pandemic [7,8].

The moderate-to-severe course of eczema results in low treatment satisfaction among patients. This dissatisfaction often brings along a low quality of life [9]. Patients have reported that eczema symptoms limit daily routine behaviors and activities, and that they avoid social touch and contact [10]. The social avoidance behaviors and tendencies of patients with eczema, the majority of whom are women, may be associated with difficulties in sexual function, an important physiological and psychological need. Sexual dysfunction is highly sensitive to psychological and social problems [11]. A limited number of studies have addressed sexual dysfunction in patients with eczema within the general population. A systematic review reported that studies on individuals with dermatological diseases focused more on psychological comorbidities such as anxiety and depression; conversely, sexual dysfunction has been very little investigated. The same systematic review emphasized that the literature has predominantly investigated sexual dysfunction in men with dermatological disorders [12]. The present study complements existing research by examining sexual function alongside medication-related motivation, knowledge, and perceived spousal support in women with eczema.

Sexual experiences may involve desire, physiological arousal, orgasm, and satisfaction, although these do not necessarily occur together or in a fixed sequence [13]. Self-confidence is crucial for exhibiting good sexual performance from psychological and social perspectives [14]. A positive body image may contribute to a woman’s sexual well-being. Women with eczema may feel uncomfortable having affected skin touched or may experience pain. Understanding how women with eczema perceive support from their partners may help contextualize their sexual experiences. Accordingly, this study examined perceived spousal support alongside sexual function and medication-related motivation and knowledge [15]. Good spousal support provided to women with eczema may be associated with better sexual functioning and medication adherence. Indeed, since the symptoms of moderate and severe eczema can persist despite medication adherence, they carry the potential to reduce patients’ resilience to medication non-adherence. In this study, we investigated sexual function, perceived spousal support, and medication-related motivation and knowledge in women with eczema.

## 2. Method

### 2.1. Research Design and Participants

Our study aimed to define the medication-related motivation and knowledge of female patients with eczema, as well as to determine their sexual dysfunction and perceived spousal support, and to examine the relationship between these concepts. The nature of this study is descriptive and cross-sectional. Data were collected between 1 April 2025 and 31 January 2026. Purposive sampling was employed as the primary sampling strategy. All women attending routine follow-up in the dermatology unit during this period who met the inclusion criteria were invited to participate. No a priori sample size calculation was performed; all eligible women who agreed to participate during the data collection period were included. With 218 participants, the study had 80% power to detect a correlation of r = 0.19 (two-sided α = 0.05). There were no missing responses, so no scoring rule for incomplete responses was needed, and all analyses used N = 218. The questionnaires were self-completed by the participants. Following institutional ethical approval, the research was carried out with the participation of female patients with eczema who agreed to participate. Data collection was performed in the dermatology unit of a private hospital in İzmir, Turkey. Women with eczema, whose written informed consent was obtained, completed the questionnaires within a 15-min period. The inclusion and exclusion criteria for the sample in our study are presented below.

### 2.2. Inclusion and Exclusion Criteria

The inclusion criteria for this study were: (i) being regularly followed up in the dermatology unit where the study was conducted, (ii) having been diagnosed with eczema at least 6 months ago and possessing medications prescribed by a physician, (iii) being female and aged 18 years and older, with no communication or language barriers, (iv) being married or having an emotional relationship of at least 6 months, and sharing the same house with a partner or spouse for at least 6 months.

The exclusion criteria for this study were: (i) having a dermatological disease other than eczema, (ii) being treated in a different hospital or not having regular follow-up checkups, (iii) patients under 18 years of age, individuals who do not speak or read/write Turkish, those with communication-language barriers, and male individuals, (iv) patients who do not have a partner/spouse, are divorced, live in separate houses, or have been together for less than 6 months were excluded from the sample.

The diagnosis of eczema was established by dermatologists and confirmed through clinical records. Although the use of antihistamines was not an inclusion criterion, all eligible women were using antihistamines. Participants completed the questionnaire regarding medication use by taking into account all eczema medications that had been prescribed to them.

### 2.3. Data Collection Tools

A personal information form, the Modified Morisky Adherence Scale (MMAS), the Spousal Support (SS) Scale, and the Arizona Sexual Experience Scale (ASEX) were used as data collection tools in the study. Information regarding the scales is presented below.

#### 2.3.1. Personal Information Form

The personal information form is an information form in which the demographic characteristics and health-related data of women with eczema are recorded. The form also included a single question, “Do you receive support from your spouse?” (yes/no), which referred to general spousal support without a specified recall period. Chronic disease (present/absent) and menopausal status (yes/no) were self-reported; the types of chronic disease were not recorded. Menopausal status was not defined by clinical or hormonal criteria, and perimenopausal status was not assessed separately. Sexual intercourse frequency was assessed with three options (“never”, “1–3 times a week”, “5–7 times a week”) without a specified recall period. Sexual dysfunction was self-reported with the question “Do you have sexual dysfunction?” (yes/no), which referred to the participant’s own perception rather than a previous diagnosis.

#### 2.3.2. Modified Morisky Adherence Scale

In this study, the Modified Morisky Scale 6 was used to evaluate the participants’ knowledge and motivation levels related to adherence to medication treatment [16,17,18]. The Turkish validity and reliability study (six-item) was conducted by Vural, Acar, Topsever, and Filiz. The scale consists of 6 items, and the questions are answered as “yes/no”. In the scale, items 1, 2, and 6 evaluate the motivation level, while items 3, 4, and 5 evaluate the knowledge level. In scoring, a “yes” response to items 2 and 5 is evaluated as 1 point, and a “no” response as 0 points; for the other questions, a “yes” response is scored as 0 points, and a “no” response as 1 point. The total score ranges from 0 to 6, and each subscale ranges from 0 to 3. Higher scores indicate higher medication-related motivation and knowledge. A total score of 0 or 1 in the motivation and knowledge subdimensions indicates a low level, while a score above 1 indicates a high level. The scale is a short, easily applicable measurement tool that can be used to evaluate adherence to long-term medication treatment in chronic diseases [19]. In this study, the KR-20 internal consistency coefficient of the six-item Modified Morisky Scale was calculated as 0.861.

#### 2.3.3. Spousal Support Scale

The Spousal Support (SS) Scale was developed by Yıldırım in 2004. It is a 27-item, 3-point Likert-type scale (Applies to me = 3 points, Partially applies to me = 2 points, Does not apply to me = 1 point). The SS Scale consists of a total of 4 subdimensions: emotional support (9 items), financial help and informational support (7 items), appreciation support (8 items), and social interest support (3 items). The reverse-scored questions in the scale are items 10, 20, and 24. The minimum and maximum scores obtained from the scale are 27 and 81 points, respectively. An increase in the mean score indicates an increase in the person’s perceived spousal support. In the original scale, the Cronbach’s alpha internal consistency coefficient was found to be 0.95 [20]. In our study, the scale’s Cronbach’s alpha internal consistency coefficient was found to be 0.98.

#### 2.3.4. Arizona Sexual Experience Scale

The Arizona Sexual Experience Scale (ASEX) has both male and female forms; the female form was used in this study. Developed by McGahuey et al. [21] in 2000, the ASEX was adapted into Turkish by Soykan [22] (2004), who reported a Cronbach’s alpha coefficient of 0.90. The ASEX is a five-item self-report scale in which each item is scored from 1 to 6. The female form assesses sexual desire, arousal, vaginal lubrication, the ability to achieve orgasm, and satisfaction derived from orgasm. Total scores range from 5 to 30. Higher scores indicate greater sexual function difficulties. In this study, scores above 11 (>11) were considered positive, based on Soykan’s Turkish validation study involving hemodialysis patients, in which scores ≤11 are considered negative [22]. Positive scores were interpreted as a screening result for potential sexual dysfunction rather than a clinical diagnosis. Since this cutoff value was derived from a different clinical population, caution should be exercised regarding its applicability to women with eczema. Therefore, the positive screening rate was also calculated using the original criteria of McGahuey et al. (total score ≥ 19, any item score ≥ 5, or three or more item scores ≥ 4) [21]. Participants rated their experience over the past week. The ASEX was selected because it is brief, has a validated Turkish version, and places a low burden on respondents in a routine outpatient setting. In our study, the Cronbach’s alpha value was found to be 0.96.

### 2.4. Statistical Analyses

In the evaluation of statistical outputs, a 95% confidence interval and a significance level of *p* < 0.05 were taken as a basis. After coding the obtained data into Excel, they were analyzed using IBM SPSS Statistics for Windows, Version 27.0, JASP version 0.19.0. Descriptive tests (number, percentage, and mean) were used in the evaluation of demographic data. The normality of the scale scores was examined using skewness and kurtosis values. The skewness and kurtosis values of the ASEX (0.044 and −1.592), MMAS (0.480 and −1.195), and SS (−0.020 and −1.870) scores were within the range of −2 to +2, suggesting that the distributions were approximately normal. An Independent samples t-test was used to compare numerical data between two independent groups, and a one-way analysis of variance (ANOVA) was used to compare more than two independent groups. Pearson correlation analysis was performed to determine the relationship between scale scores, and multiple regression analysis was performed to determine the effect. In addition, Spearman correlations and non-parametric tests (Mann–Whitney U and Kruskal–Wallis) were performed as sensitivity analyses.

Three-step hierarchical multiple linear regression was performed with ASEX total score as the dependent variable. Model 1 included MMAS and SS total scores. Model 2 additionally included age, menopausal status, chronic disease status, and antihistamine use duration. These covariates were selected a priori as established determinants of female sexual function (age, menopause, chronic disease) and as a medication-related characteristic (treatment duration). Other variables that differed in ASEX scores were not included because they overlapped conceptually with the outcome (self-reported sexual dysfunction and the yes/no spousal support item) or were associated with the selected covariates (antihistamine frequency, side effects, employment, residence, and income), which would have increased multicollinearity. Model 3 additionally included sexual intercourse frequency as a sensitivity analysis because of its conceptual overlap with sexual functioning; it was not included in the primary model because it may be a correlate or consequence of sexual function rather than an independent determinant. Categorical predictors were dummy-coded. Model performance was evaluated using R^2^, adjusted R^2^, and changes in R^2^. Multicollinearity was assessed using tolerance and variance inflation factors.

## 3. Results

Of the women participating in the study, 40.8% live in a village, and 92.2% have social security. Additionally, 66.5% of the women are actively working, and 49.5% have an income equal to their expenses. According to their health histories, 57.8% of the participants do not have a chronic disease, and 83.5% are premenopausal. All participants were utilizing antihistamines, with 71.6% administering the medication twice daily. Furthermore, 67.9% of the participants take the medication during daytime hours; 58.3% have been using it for 2–4 years, 18.8% reported experiencing side effects, and 81.2% reported experiencing no side effects. Regarding sexual life, 51.4% of the participants stated that they had sexual intercourse 1–3 times a week. While 94.5% of the women self-reported that they did not have sexual dysfunction, the positive screening rate for potential sexual dysfunction according to the ASEX cutoff score was found to be 59.2%. Moreover, 56.9% of the participants answered “no” to the single yes/no question on spousal support (Table 1).

When the scale score means of the 218 individuals participating in the study were examined, the Spousal Support Scale (SS) mean was 53.34 ± 20.39, revealing that they perceived a moderate level of spousal support. The Arizona Sexual Experience Scale (ASEX) mean was 16.19 ± 8.94 and the positive screening rate for potential sexual dysfunction in the sample was found to be 59.2% (n = 129) according to the Turkish cutoff and 50.5% (n = 110) according to the original McGahuey criteria. All 12 women who self-reported sexual dysfunction screened positive according to the Turkish cutoff, compared with 117 of 206 women (56.8%) who did not. The Modified Morisky Adherence Scale (MMAS) mean was found to be 2.53 ± 2.21 (Subdimensions: Motivation score mean 1.20 ± 1.22 and Knowledge score mean 1.33 ± 1.12) (Table 1). Low motivation was found in 141 participants (64.7%) and low knowledge in 130 (59.6%) (Table 1).

**Figure 1:**
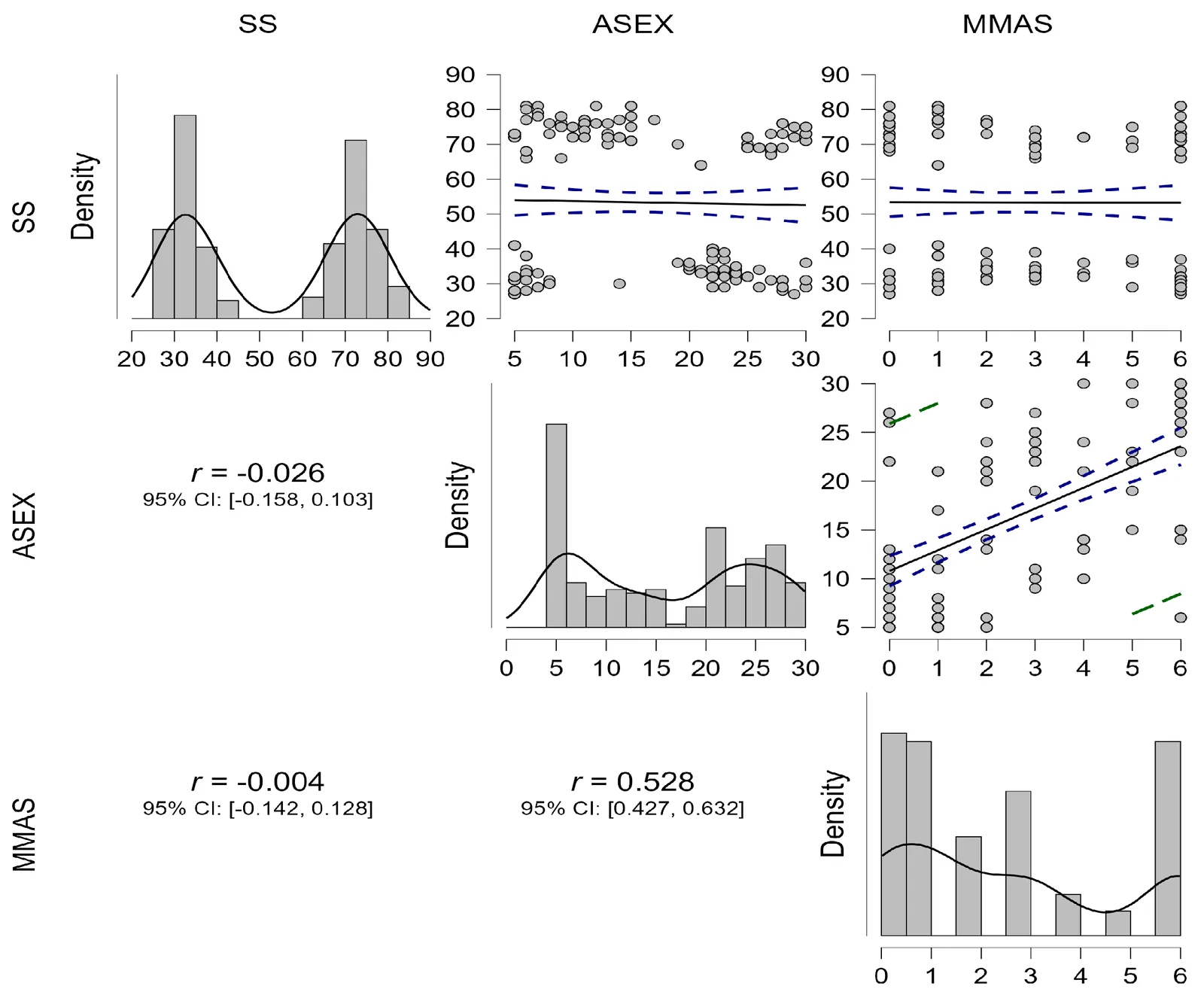
Distributions and Pairwise Relationships of Spousal Support Scale (SS), Arizona Sexual Experience Scale (ASEX), and Modified Morisky Adherence Scale (MMAS) Scores: Distributions, Pairwise Scatterplots, and Pearson Correlation Coefficients. **Note**. Diagonal panels show histograms with kernel density curves. Upper panels show scatterplots with the linear regression line (solid black), its 95% confidence band (blue dashed lines), and the 95% prediction interval (green dashed lines). Lower panels show Pearson correlation coefficients with 95% confidence intervals (N = 218).

When the relationships between the scales were examined by Pearson correlation analysis, a positive, moderate, and statistically significant relationship was found between the Arizona Sexual Experience Scale (ASEX) and Modified Morisky Adherence Scale (MMAS) scores (r = 0.528; *p* < 0.001). Higher MMAS scores were associated with higher ASEX scores. On the other hand, no statistically significant relationship was found between Spousal Support Scale (SS) scores and ASEX (r = −0.026; *p* = 0.704) and MMAS (r = −0.004; *p* = 0.958) scores (*p* > 0.05) (Table 1; Figure 1). ASEX scores were found to be positively correlated with scores on both the motivation (r = 0.568; *p* < 0.001) and knowledge (r = 0.426; *p* < 0.001) subdimensions. The single yes/no spousal support question (coded yes = 1, no = 0) was not correlated with the Spousal Support Scale total score (r = 0.016, *p* = 0.812). As a sensitivity analysis, the correlations were also examined using Spearman’s rank correlation, which yielded similar results (ASEX – MMAS ρ = 0.556, ASEX – motivation ρ = 0.629, ASEX – knowledge ρ = 0.428, all *p* < 0.001; ASEX – SS ρ = 0.007, *p* = 0.923).

A three-stage hierarchical linear regression analysis was conducted, with the total ASEX score as the dependent variable. In the first stage, MMAS and SS scores were included in the model. This model was significant (F(2, 215) = 41.63; *p* < 0.001) and explained 27.9% of the variance in ASEX scores (R^2^ = 0.279). A positive association was found only between MMAS scores and ASEX scores (β = 0.528; *p* < 0.001).

In the second stage, age, menopausal status, chronic disease, and the duration of antihistamine use were added to the model. These variables significantly increased the proportion of explained variance (ΔR^2^ = 0.234; ΔF(4, 211) = 25.42; *p* < 0.001), and the overall model explained 51.4% of the variance (R^2^ = 0.514; adjusted R^2^ = 0.500; F(6, 211) = 37.13; *p* < 0.001). This model was adopted as the primary model. The association between MMAS and ASEX scores remained significant but weakened considerably compared to the first stage (β = 0.173; *p* = 0.038). Older age was associated with higher ASEX scores (β = 0.345; *p* = 0.001), while a 2–4 years duration of antihistamine use was associated with lower ASEX scores (β = −0.272; *p* < 0.001). Chronic disease (β = −0.142; *p* = 0.056) and menopausal status (β = −0.005; *p* = 0.945) were not found to be significant in this model.

In the third stage, frequency of sexual intercourse was added to the model as a sensitivity analysis. The explained variance increased by an additional 19.1 percentage points (ΔR^2^ = 0.191; ΔF(2, 209) = 67.82; *p* < 0.001), and the final model accounted for 70.5% of the variance (R^2^ = 0.705; adjusted R^2^ = 0.694; F(8, 209) = 62.44; *p* < 0.001). In this model, not having reached menopause (β = −0.269; *p* < 0.001), antihistamine use for 2–4 years (β = −0.147; *p* = 0.001), and sexual intercourse 5–7 times per week (β = −0.464; *p* < 0.001) were associated with lower ASEX scores. The association with MMAS scores was no longer significant (β = 0.078; *p* = 0.237). SS scores showed a borderline negative association (β = −0.079; *p* = 0.049). This association was not observed in the first two models; therefore, it should be interpreted with caution. Because intercourse frequency may be a correlate or consequence of sexual difficulties, the attenuation of the MMAS association after its inclusion does not establish mediation.

Holding other variables constant, the unstandardized coefficients are as follows: It was shown that the ASEX scores of premenopausal women were 6.469 points lower compared to those of postmenopausal women. Individuals who had used antihistamines for 2–4 years had scores 2.655 points lower than those who had used them for 1 year, while women who reported engaging in sexual intercourse 5–7 times per week had scores 8.844 points lower than those who reported no sexual activity. In the final model, VIF values ranged from 1.13 to 6.11. Although all values were below 10, caution should be exercised when interpreting the coefficients for age (5.51) and sexual intercourse 5–7 times per week (6.11), as their VIF values exceeded 5. The coefficient for menopausal status also showed a notable change between the second and third models. Given the close relationship between menopausal status and age, this variable should not be interpreted in isolation (Table 2).

When the data in Table 3 are examined, it was found that the participants’ Arizona Sexual Experience Scale (ASEX) score means showed statistically significant differences according to all variables except social security and antihistamine use time (*p* < 0.05). Evaluated in terms of demographic variables, a significant difference was found between ASEX score means according to the place of residence (*p* = 0.010). As a result of Bonferroni post hoc analysis, the ASEX score mean of those living in the city center was 18.61 ± 9.23, which is higher than the mean of 13.98 ± 8.98 for those living in the districts. In terms of employment status, the ASEX score mean of unemployed participants (19.34 ± 9.72) was significantly higher than that of employed participants (14.61 ± 8.12) (*p* < 0.001). A significant difference was also found according to income status (*p* = 0.040). As a result of Bonferroni post hoc analysis, the score mean of those whose income is equal to their expenses was 17.48 ± 8.79, which is higher than that of those whose income is less than their expenses (13.15 ± 8.78). When health status variables were evaluated, the ASEX score mean of participants with chronic diseases (22.08 ± 7.16) was statistically significantly higher than that of those without chronic diseases (11.90 ± 7.59) (*p* < 0.001). Similarly, the score mean of postmenopausal women (25.94 ± 5.21) was significantly higher than that of premenopausal women (14.26 ± 8.25); the mean of those self-reporting sexual dysfunction (28.50 ± 1.09) was higher than that of those who did not (15.48 ± 8.68); and the mean of those without spousal support (22.92 ± 5.10) was significantly higher than that of those with spousal support (7.32 ± 3.54) (all *p* < 0.001). Looking at medication use characteristics, those using medication twice a day (19.03 ± 8.18) had higher scores compared to those using it once a day, and those using the medication for 1 year (20.53 ± 8.54) had higher scores compared to those using it for 2–4 years (*p* < 0.001). In addition, the mean score of the group experiencing no side effects (17.85 ± 8.63) was found to be significantly higher than that of the group experiencing side effects (9.05 ± 6.52) (*p* < 0.001). In the frequency of sexual intercourse variable, as a result of pairwise comparisons with Bonferroni correction, it was determined that all of the “5–7 times a week” (6.37 ± 2.91), “1–3 times a week” (20.12 ± 6.59), and “never” (23.54 ± 6.61) groups differed significantly from each other. Antihistamine use time (daytime/nighttime) did not create a statistically significant difference in ASEX score means (*p* = 0.100) (Table 3). Non-parametric tests (Mann–Whitney U and Kruskal–Wallis) yielded the same conclusions, except for income status (*p* = 0.073). These comparisons were exploratory. After Holm correction, all differences remained significant except income status.

## 4. Discussion

This study contributes to the literature by addressing sexual function, medication adherence, and perceived spousal support together in women with eczema. In this study, the ASEX scores obtained by women with eczema who had comorbidities, were menopausal, and had no spousal support were above the cutoff value (>11), and these differences were statistically significant compared to others (all *p* < 0.001). In this study, 83.5% of the women with eczema were not menopausal, and 59.2% screened positive for potential sexual dysfunction. A previous study reported that eczema provides a disadvantage in terms of sexual health and romantic relationships [23]. A narrative review stated that eczema, particularly affecting the genital area, impairs quality of life and sexual health. The same study reported that eczema weakens the perception of sexual satisfaction and appearance in women [24]. Another study reported that nearly 25% of women with eczema in a visible area experienced various difficulties in working life due to their eczema [25]. In this study, 40.8% of the women with eczema lived in a village, 66.5% were actively working, and 49.5% had a moderate income.

While only 5.5% of the participants reported experiencing a sexual problem, 59.2% screened positive according to the ASEX scale. All women who reported a sexual problem also screened positive; however, the vast majority of those who screened positive did not report any problems. There are several factors that may explain this discrepancy. First, the threshold value was derived from hemodialysis patients and may have been set too low for women with eczema, which could have led to an inflated prevalence rate. Indeed, when the original McGahuey criteria were applied, the prevalence rate dropped to 50.5%. Second, the ASEX measures sexual function difficulties; women may not perceive or define these conditions as a “problem.” Third, as sexuality remains a sensitive topic in Turkish society, women might be reluctant to disclose sexual issues when responding to a single, direct question. The ASEX does not assess sexual distress, which is required for a clinical diagnosis of sexual dysfunction; women may experience difficulties without being distressed by them. Accordingly, questionnaire-detected sexual function difficulties, subjective perceptions of a sexual problem, and clinically diagnosed sexual disorders should be distinguished. Therefore, ASEX results should be interpreted as screening findings rather than clinical diagnoses.

Secondly, this study reveals the perceived spousal support of women with eczema. In this context, 56.9% of women with eczema reported not receiving spousal support in response to a single yes/no question. A previous study determined that there was a dramatic increase in the risk of atopic eczema in the first 3 months and a modest increase in the subsequent 3 years following partner bereavement [26]. Acute stress can cause eczema to appear for the first time or relapse from remission. The relationship between eczema and psychological events has been previously reported in several studies [27,28,29]. On the other hand, this study found no association between the total score on the multi-item Spousal Support Scale and scores on the ASEX (Arizona Sexual Experiences Scale). In contrast, a single “yes/no” question regarding partner support showed a strong association with ASEX scores; however, these two measures of support were not found to be correlated with each other. A lack of adequate partner support can exacerbate psychological distress-a well-known factor that can trigger or worsen eczema lesions. Two previous studies have shown that partner support improves sexual dysfunction in women who have undergone liver or kidney transplants [13,30]. Nevertheless, the discrepancy between these two measures of support in our sample could not be explained by the available data; this makes it difficult to draw any conclusions regarding the relationship between partner support and sexual function in women with eczema.

Thirdly, this study assessed medication-related motivation and knowledge in women with eczema. Medications used in the treatment of eczema in the literature are listed as topical corticosteroids alone or in combination with systemic acyclovir therapy, corticosteroids and calcineurin inhibitors, antihistamine drugs and aminophylline, tumor necrosis factor (TNF)-α inhibitors. antivirals. and antibiotics [31,32,33,34,35,36,37]. The success rate of eczema treatment is affected by various factors; these may often be related to not avoiding conditions that cause skin irritation (detergent, solvents, and sweat, etc.), the continuation of high stress exposure, the patient’s problems in managing a heavy care burden along with comorbidities, and low medication-related motivation and knowledge due to various reasons (expensive medications, forgetting to use, skipping doses, etc.) [28,38]. In a study conducted with the participation of 237 psoriasis patients, the scores obtained according to the MMAS scale-8 were reported as 5.2 for oral medications and 4.3 for topical medications [39]. In our study, the mean score obtained from the MMAS scale-6 was 2.53 ± 2.21. The MMAS-8 scores reported in the previous study are not directly comparable with the six-item Modified Morisky scores used in our study. In our study, higher medication-related motivation and knowledge scores were associated with greater sexual function difficulties. In the hierarchical regression, this association remained significant after adjustment for age, menopausal status, chronic disease, and antihistamine use duration, but it was considerably weaker (B = 0.700, *p* = 0.038). After additionally including intercourse frequency, the association was no longer statistically significant (B = 0.316, *p* = 0.237). This additional adjustment requires cautious interpretation because sexual intercourse frequency conceptually overlaps with aspects of sexual function assessed by ASEX. Furthermore, higher MMAS scores do not directly demonstrate greater medication exposure. Since antihistamine names, doses, and actual exposure were not recorded, medication-specific effects could not be determined, and causal conclusions cannot be drawn. Therefore, this association should not be interpreted as indicating that greater adherence worsens sexual function.

### Limitations

This study has several limitations. First, the cross-sectional design does not allow for determining the direction and causality of relationships between variables. The single-center study and the use of purposive sampling may also limit the generalizability of the findings to different groups. The number of women who were approached but declined to participate was not recorded, and no sample size calculation was performed prior to the study. The assessment of sexual function, motivation and knowledge regarding medication use, and perceived partner support-all conducted using self-report scales—may have been subject to recall bias and social desirability bias, particularly given the sensitive nature of the subject matter, such as sexual health. Furthermore, since the severity of eczema and the location of lesions were not assessed using validated disease-specific tools, it was not possible to examine in detail whether sexual dysfunction differed according to disease severity or affected area. Although participants had previously been diagnosed with eczema, the diagnostic criteria and eczema subtypes were not recorded. Vulvar or anogenital involvement may affect sexual function directly through pruritus, irritation, and pain, whereas non-genital eczema may act through body image and psychological distress; this distinction could not be examined. In addition, the ASEX does not assess dyspareunia, genital pain, or sexual distress, and instruments with a pain domain, such as the Female Sexual Function Index, would provide a broader assessment in future studies. Furthermore, information regarding chronic illnesses and menopausal status was obtained based on the individuals’ own reports, and the specific types of chronic illnesses were not recorded. Data concerning education level, duration of the relationship, and reproductive characteristics were not collected.

The lack of objective verification of medication adherence and the inclusion of women using antihistamines in the sample made it difficult to evaluate the effects of different treatment approaches separately. Antihistamine names, doses, and concomitant topical or systemic treatments were not recorded. Consequently, possible medication-specific effects could not be assessed separately from disease-related factors. Moreover, the Modified Morisky Scale assessed medication-related motivation and knowledge rather than actual drug exposure. In addition, because antihistamines differ in their sedative and anticholinergic properties, treatment-related effects could not be assessed.

Participants who reported not engaging in sexual intercourse responded to the ASEX items themselves; however, the specific experiences upon which they based their responses regarding orgasm and satisfaction were not examined. This creates ambiguity regarding the interpretation of these responses. Sexual intercourse frequency conceptually overlaps with various aspects of sexual function assessed by the ASEX; therefore, caution is required when interpreting its inclusion in the additional regression model. In addition, the frequency question offered only three options without a specified recall period and did not cover all possible frequencies. When the 35 women who reported no intercourse were excluded, the association between MMAS and ASEX scores in the primary model was similar (B = 0.777, *p* = 0.033). Sexual inactivity should not be equated with dysfunction.

The absence of a significant correlation between the SS total score and the binary (yes/no) question regarding partner support indicates limited concordance between these assessments in the current sample. The reasons for this lack of concordance could not be determined from the available data.

Additionally, in the absence of a control group, the findings cannot be directly attributed to eczema or the use of antihistamines; the observed associations should be considered exploratory and specific to this sample. When interpreting the results, one must account for potential unmeasured confounding variables—such as depression and anxiety, sleep disturbances, relationship quality, vulvovaginal dryness, dyspareunia, genitourinary syndrome of menopause, gynecological disorders, pregnancy, the postpartum period, breastfeeding, hormonal contraception, and menopausal hormone therapy—as well as medications that may influence sexual function, such as antidepressants.

## 5. Conclusions

This study shows that potential sexual dysfunction appears to be an important clinical concern in women with eczema who use antihistamines. Only 5.5% of participants reported a sexual problem, whereas 59.2% screened positive for potential sexual dysfunction according to the ASEX cutoff. This discrepancy suggests that self-reported sexual problems and screening results may differ and supports attention to sexual health during clinical assessment.

Research findings revealed that ASEX scores showed significant differences according to place of residence, employment status, income status, presence of chronic disease, menopausal status, frequency of antihistamine use, duration of antihistamine use, status of side effects due to antihistamine use, frequency of sexual intercourse, self-report of sexual dysfunction, and status of spousal support. The higher ASEX scores in women who have a chronic disease, are in the menopausal period, are unemployed, and report a lack of spousal support suggest that sexual function is a multidimensional construct associated with biological, psychosocial, and relational factors.

MMAS scores remained positively associated with ASEX scores after adjustment for age, menopausal status, chronic disease, and antihistamine use duration, although the association was weak (B = 0.700, *p* = 0.038), and it was no longer statistically significant after additionally including intercourse frequency (B = 0.316, *p* = 0.237). In contrast, the Spousal Support Scale total score did not show a significant relationship with ASEX in correlation and regression analyses. However, in group comparisons made according to the self-report of spousal support presence/absence, the significantly higher ASEX scores found in women who reported a lack of spousal support indicate that the measurement method and clinical meaning of the spousal support variable should be interpreted more carefully. Therefore, the relationship of perceived spousal support with sexual function in women with eczema should be examined with more detailed and multidimensional measurement approaches in future studies.

These findings support considering sexual health alongside dermatological symptoms in women with eczema; however, the single-center, cross-sectional design limits generalizability and does not establish causal relationships. Sexual function, menopausal status, accompanying chronic diseases, medication-related motivation and knowledge, medication use characteristics, and perceived spousal support should be addressed together in clinical evaluations. It is recommended that healthcare professionals evaluate women with eczema with a holistic approach, address sexual health-related issues in a safe and open communication environment, and provide counseling or referral services when necessary. Future studies to be conducted with larger samples, control groups, and longitudinal designs will contribute to a clearer elucidation of the relationships between eczema, antihistamine use, medication-related motivation and knowledge, spousal support, and female sexual function.

## Figures and Tables

**Table 1:** Sociodemographic Characteristics, Medication Use Habits, and Sexual Function Status of Women Using Antihistamines.

Variable	Category	n	%
Age (years), mean ± SD (range) 34.21 ± 8.50 (18–53)			
Place of residence	City	46	21.1
	District	83	38.1
	Village	89	40.8
Social Security	Yes	201	92.2
	No	17	7.8
Employment Status	Employed	145	66.5
	Unemployed	73	33.5
Income Status	Income < Expenses	34	15.6
	Income = Expenses	108	49.5
	Income > Expenses	76	34.9
Chronic Disease	Yes	92	42.2
	No	126	57.8
Menopausal Status	Yes	36	16.5
	No	182	83.5
Antihistamine Use Frequency	Once a day	62	28.4
	Twice a day	156	71.6
Antihistamine Use Time	Daytime	148	67.9
	Nighttime	70	32.1
Side Effects Due to Antihistamine Use	Yes	41	18.8
	No	177	81.2
Duration of Use	1 Year	91	41.7
	2–4 Years	127	58.3
Frequency of Sexual Intercourse	Never	35	16.1
	1–3 times a week	112	51.4
	5–7 times a week	71	32.6
Sexual Dysfunction Self-Report	Yes	12	5.5
	No	206	94.5
Spousal Support	Yes	94	43.1
	No	124	56.9
Spousal Support Scale: 53.34 ± 20.39 Arizona Sexual Experience Scale (ASEX): 16.19 ± 8.94 Modified Morisky Adherence Scale (MMAS): 2.53 ± 2.21 Motivation: 1.20 ± 1.22; Knowledge: 1.33 ± 1.12			

ASEX–MMAS, r = 0.528; *p* < 0.001; ASEX–SS, r = −0.026; 0.704; MMAS–SS, r = −0.004; 0.958.

**Table 2:** Hierarchical Multiple Linear Regression Analysis of Factors Associated with ASEX Scores.

Model and Variables	B	SE	95% CI	β	t	*p*	VIF
**Model 1**							
Constant	11.349	1.569	8.257 to 14.442	-	7.23	<0.001	-
MMAS	2.134	0.234	1.673 to 2.596	0.528	9.11	<0.001	1.00
SS	−0.011	0.025	−0.061 to 0.040	−0.024	−0.41	0.679	1.00
**Model 2**							
Constant	7.731	4.847	−1.824 to 17.286	-	1.60	0.112	-
MMAS	0.700	0.335	0.040 to 1.361	0.173	2.09	0.038	2.98
SS	−0.024	0.022	−0.068 to 0.020	−0.055	−1.09	0.279	1.10
Age (years)	0.363	0.108	0.151 to 0.575	0.345	3.38	0.001	4.53
Menopausal status (No)	−0.121	1.740	−3.550 to 3.308	−0.005	−0.07	0.945	2.27
Chronic disease (No)	−2.563	1.335	−5.195 to 0.068	−0.142	−1.92	0.056	2.37
Duration of antihistamine use (2–4 years)	−4.929	0.978	−6.856 to −3.001	−0.272	−5.04	<0.001	1.27
**Model 3**							
Constant	23.368	4.395	14.703 to 32.032	-	5.32	<0.001	-
MMAS	0.316	0.266	−0.209 to 0.841	0.078	1.19	0.237	3.07
SS	−0.035	0.018	−0.069 to −0.000	−0.079	−1.98	0.049	1.13
Age (years)	0.071	0.093	−0.112 to 0.254	0.067	0.76	0.446	5.51
Menopausal status (No)	−6.469	1.682	−9.785 to −3.152	−0.269	−3.85	<0.001	3.47
Chronic disease (No)	−0.136	1.065	−2.236 to 1.965	−0.008	−0.13	0.899	2.46
Duration of antihistamine use (2–4 years)	−2.655	0.802	−4.235 to −1.074	−0.147	−3.31	0.001	1.39
Frequency of sexual intercourse (1–3 times/week)	2.652	1.494	−0.292 to 5.597	0.148	1.78	0.077	4.96
Frequency of sexual intercourse (5–7 times/week)	−8.844	1.769	−12.331 to −5.357	−0.464	−5.00	<0.001	6.11

**Note.** 218. Dependent variable: ASEX total score. B, unstandardized coefficient; SE, standard error; CI, confidence interval; β, standardized coefficient; VIF, variance inflation factor. Model 1: R^2^ = 0.279, adjusted R^2^ = 0.272, F(2, 215) = 41.63, *p* < 0.001. Model 2: R^2^ = 0.514, adjusted R^2^ = 0.500, ΔR^2^ = 0.234, ΔF(4, 211) = 25.42, *p* < 0.001. Model 3: R^2^ = 0.705, adjusted R^2^ = 0.694, ΔR^2^ = 0.191, ΔF(2, 209) = 67.82, *p* < 0.001. Reference categories: menopausal status = yes; chronic disease = yes; duration of antihistamine use = 1 year; frequency of sexual intercourse = never.

**Table 3:** Comparison of Arizona Sexual Experience (ASEX) Scores According to Medication Use Habits, Sexual Function Status, and Demographics.

Variables		(%)	$\bar{X}$ ± SD	Test	*p*
Place of residence	City (1)	21.1	18.61 ± 9.23	4.755 **^b^**	0.010 * 1 > 2
	District (2)	38.1	13.98 ± 8.98		
	Village (3)	40.8	17.01 ± 8.39		
Social Security	Yes	92.2	16.10 ± 8.94	−0.528 **^a^**	0.598
	No	7.8	17.29 ± 9.25		
Employment Status	Employed	66.5	14.61 ± 8.12	−3.582 **^a^**	<0.001 *
	Unemployed	33.5	19.34 ± 9.72		
Income Status	Income < Expenses (1)	15.6	13.15 ± 8.78	3.260 **^b^**	0.040 * 2 > 1
	Income = Expenses (2)	49.5	17.48 ± 8.79		
	Income > Expenses (3)	34.9	15.72 ± 8.98		
Chronic Disease	Yes	42.2	22.08 ± 7.16	10.015 **^a^**	<0.001 *
	No	57.8	11.90 ± 7.59		
Menopausal Status	Yes	16.5	25.94 ± 5.21	10.998 **^a^**	<0.001 *
	No	83.5	14.26 ± 8.25		
Antihistamine Use Frequency	Once a day	28.4	9.05 ± 6.51	−9.464 **^a^**	<0.001 *
	Twice a day	71.6	19.03 ± 8.18		
Antihistamine Use Time	Daytime	67.9	15.51 ± 8.67	−1.652 **^a^**	0.100
	Nighttime	32.1	17.64 ± 9.40		
Side Effects Due to Antihistamine Use	Yes	18.8	9.05 ± 6.52	−7.289 **^a^**	<0.001 *
	No	81.2	17.85 ± 8.63		
Duration of Use	1 Year	41.7	20.53 ± 8.54	6.626 **^a^**	<0.001 *
	2–4 Years	58.3	13.09 ± 7.91		
Frequency of Sexual Intercourse	Never	16.1	23.54 ± 6.61	163.313 **^b^**	<0.001 *
	1–3 times a week	51.4	20.12 ± 6.59		
	5–7 times a week	32.6	6.37 ± 2.91		
Sexual Dysfunction Self-Report	Yes	5.5	28.50 ± 1.09	19.118 **^a^**	<0.001 *
	No	94.5	15.48 ± 8.68		
Spousal Support	Yes	43.1	7.32 ± 3.54	−26.635 **^a^**	<0.001 *
	No	56.9	22.92 ± 5.10		

* *p* < 0.05. a = Independent t test, b = One Way ANOVA.

## Data Availability

The datasets generated and analyzed during the current study are not publicly available due to patient confidentiality, the conditions of the ethics approval, and the data-protection policy of İzmir Metropol Hospital, where the data were collected. The anonymized dataset is available from the corresponding author upon reasonable request, subject to permission from the hospital.
